# Early-Onset Osteoarthritis originates at the chondrocyte level in Hip Dysplasia

**DOI:** 10.1038/s41598-020-57431-x

**Published:** 2020-01-17

**Authors:** Paula A. Hernandez, Joel Wells, Emiliya Usheva, Paul A. Nakonezny, Zahra Barati, Roberto Gonzalez, Layla Kassem, Frances M. D. Henson

**Affiliations:** 1 Department of Orthopaedic Surgery, University of Texas Southwestern Medical Center, Dallas, TX 75390 USA; 2 Department of Population and Data Sciences, Division of Biostatistics, University of Texas Southwestern Medical Center, Dallas, TX 75390 USA; 3 Division of Trauma and Orthopaedic Surgery, University of Cambridge, Cambridge, CB2 2QQ UK

**Keywords:** Integrins, Intermediate filaments, Osteoarthritis

## Abstract

Subjects with developmental dysplasia of the hip (DDH) often show early-onset osteoarthritis (OA); however, the molecular mechanisms underlying this pathology are not known. We investigated whether cellular changes in chondrocytes from OA cartilage can be detected in chondrocytes from DDH cartilage before histological manifestations of degeneration. We characterized undamaged and damaged articular cartilage from 22 participants having hip replacement surgery with and without DDH (9 DDH-OA, 12 OA-only, one femoral fracture). Tissue immunostaining revealed changes in damaged OA-only cartilage that was also found in undamaged DDH-OA cartilage. Chondrocytes *in situ* from both groups show: (i) thicker fibers of vimentin intermediate filaments, (ii) clusters of integrin α_5_β_1_, (iii) positive MMP13 staining and (iv) a higher percentage of cells expressing the serine protease HtrA1. Further characterization of the extracellular matrix showed strong aggrecan and collagen II immunostaining in undamaged DDH cartilage, with no evidence of augmented cell death by activation of caspase 3. These findings suggest that early events in DDH cartilage originate at the chondrocyte level and that DDH cartilage may provide a novel opportunity to study these early changes for the development of therapeutic targets for OA.

## Introduction

Although aging is among the various factors associated with the development of Osteoarthritis (OA)^1^, the incidence of OA has been increasing in younger populations. One study projected that the percentage of patients younger than 65 years old requiring total hip arthroplasty would increase from 41% in 2006 to 52% in 2030^2^. These younger patients have a number of etiologies, including those with developmental dysplasia of the hip (DDH). In this condition, abnormal biomechanics leads to anomalous stress distribution within the hip joint and subsequent damage to the joint tissues including articular cartilage^3–6^ and DDH is considered to be part of the congenital-biomechanical causes of OA^7^.

Chondrocytes, the cells of cartilage, are metabolically stimulated by different forms of mechanical loading including strain, shear, compression, tension, and hydrostatic loads, all of which are related to the structural and mechanical integrity of the joint. At the tissue-level, the extracellular matrix (ECM)^8^, composed mostly of collagen II and proteoglycans, gives mechanical integrity, while at the cellular-level the cytoskeleton fulfills this role, with vimentin intermediate filaments playing a major function^9,10^. Mechanical load, in turn, regulates both cytoskeleton dynamics^11,12^ and the synthesis of key ECM proteins^13,14^.

Of the key ECM components regulating mechanical signals to chondrocytes, fibronectin mediates pathways associated with cell growth and survival, for example promoting chondrocyte survival via integrin α_5_β_1_ binding^15^. However, in contrast, fibronectin fragments, produced via cleavage by proteases such as the High-Temperature Requirement protein A1 (HtrA1)^16^, activate catabolic pathways^17,18^. In OA, HtrA1 is upregulated in cartilage and synovial fluid in both human^16,19^ and murine joints^20–23^. HtrA1 was detected early in the process of cartilage degeneration^21^, and its primary function is believed to be the digestion of fibronectin and other proteins from both the pericellular matrix (PCM) and the ECM^16,19,22^. Disruption of the PCM may expose collagen II to bind the Discoidin Domain Receptor 2 (Ddr2)^21^ and consequently upregulate the matrix metalloprotease MMP13^24^, which has been most studied for its role in matrix degradation and the progression of OA^25–28^. MMP13 was found upregulated at early stages of cartilage degeneration in a rat model of DDH^29^.

While it is apparent that altered hip biomechanics leads to early-onset OA in patients with DDH, the exact mechanism by which this occurs in not known. We hypothesize that the microenvironment in histologically undamaged DDH cartilage induces OA-like phenotypic adaptations in chondrocytes. Our results revealed that in cartilage from histologically undamaged regions in hips taken from patients with DDH, the chondrocyte phenotype had marked similarities to the pathological characteristics found in damaged OA hip cartilage from patients without DDH (OA-only), which reflect the usual population of patients. We propose that significant cellular changes precede histological manifestations of OA in DDH cartilage pointing toward alterations at the chondrocyte microenvironment level as an early mechanism underlying the aetiopathogenesis of OA in hip dysplasia.

## Methods

### Ethics

All samples used in this study were obtained from patients (n = 22) having hip replacement surgery at Zale Hospital in UT Southwestern Medical Center, Dallas, TX. All patients gave informed consent to participate in this study and to collect de-identified medical waste. Study protocol, consent, and HIPAA were approved by the UT Southwestern Institutional Review Board (IRB). All methods were performed in accordance with relevant guidelines and regulations.

### Patients

Two groups of patients were identified. Twelve patients had OA with no history of DDH (40–71 y.o; 7 female, 5 male), and 9 patients had OA secondary to DDH (23–72 y.o; 5 female, 4 male). In addition, we included undamaged cartilage from the hip of one non-OA patient (femoral fracture (female, 79 y.o.) was used for the patient independent analysis. Diagnosis was made using hip radiography by an Orthopaedic surgeon with subspecialty training in hip dysplasia and hip preservation. Exclusion criteria were any history of cancer or rheumatoid arthritis.

### Experimental groups

The groups analyzed in this study were: (i) undamaged cartilage from OA-only, (ii) damaged cartilage from OA-only, (iii) undamaged cartilage from DDH-OA, and (iv) damaged cartilage from DDH-OA group. The femoral fracture case was considered undamaged cartilage and analyzed within the OA-only group.

### Cartilage harvest

Full-thickness cartilage without subchondral bone from both visibly undamaged and damaged areas of femoral heads were obtained from the femoral head using sharp dissection. Samples were fixed in 10% Neutralized Buffer Formalin and processed for paraffin embedding. Cartilage fibrillation was not homogeneous throughout the femoral heads, allowing us to collect areas with little to no damage and areas with a diverse degree of damage.

### Histological staining, scoring, and immunohistochemistry

Cartilage slides of 5 µm thickness were obtained from paraffin-embedded blocks. Slides were deparaffinized and processed for Safranin-O staining. Images of this staining were used for tissue grading with a modified Mankin score^30^. The modified Mankin score (MMS) system that we used excluded the score allocated to ‘tidemark’ as subchondral bone was not collected during sample acquisition, leading to a scale of 0 (no damage) to 13 (severe OA). Based on histological evidence of cartilage damage (reduced proteoglycan staining and surface fibrillation) we grouped samples into ‘undamaged’ (MMS ≤ 4) and ‘damaged’ cartilage (MMS > 4). This classification includes early and mild OA in the undamaged group, and moderate and severe in the damaged group^31^. Two blind investigators performed the scoring. For immunohistochemistry, antigen retrieval was not required for HtrA1, actin, caspase-3 active, and integrin α_5_β_1_ staining. For vimentin, DeCal retrieval solution was used following the manufacturer’s instructions (BioGenex, HK089-5K). For MMP13, Aggrecan and Collagen II, antigen retrieval was done by 30 min incubation with 100 mU/ml of Chondroitinase ABC (Sigma-Aldrich, C3667) in 50 mM Tris-HCl + 60 mM sodium acetate, pH 8.0, washed twice for 2 min with PBS, followed by 30 min incubation with 2 mg/ml of Hyaluronidase (Sigma-Aldrich, H3506) in PBS and 2 washes for 2 min with PBS. Both enzymatic incubations were done at 37 °C. Slides for HtrA1, actin, vimentin, integrin α_5_β_1_ and caspase 3 active were permeabilized with 0.5% Triton-X100 for 5 min. All slides were blocked for 2 hours at room temperature in blocking buffer (TBS with 5% BSA, 10% goat serum and 0.1% Tween-20), followed by overnight incubation at 4 °C with the following antibodies prepared in TBS + 5% BSA + 0.1% Tween-20: rabbit anti-HtrA1 (Abcam, ab38611; 1:100), mouse anti-actin (Abcam, ab8226; 1:100), mouse anti-integrin α_5_β_1_ (EMD Millipore, MAB1969; 1:100), rabbit anti-vimentin (Abcam, ab45939; 1:100), rabbit anti-MMP13 (Abcam, ab39012; 1:200), mouse anti-Aggrecan (Abcam, ab3778; 1:100), rabbit anti-collagen II (Abcam, ab34712; 1:400), or rabbit anti-caspase 3 active (EMD Millipore, AB3623; 1:200). Slides were washed five times for 10 min each in TBS + 0.2% Tween 20 at room temperature with gentle shaking, followed by secondary antibody goat-anti-rabbit-Alexa 555, goat-anti-mouse-Alexa 555 or goat-anti-rabbit-Alexa 488 (1:1,000; Molecular Probes) for 1 hour at room temperature in TBS + 5% BSA + 0.1% Tween-20 in the dark. After seven washes of 10 min each in TBS + 0.2% Tween 20 at room temperature with gentle shaking in the dark, samples were mounted with Diamond ProLong with DAPI. Images of the staining were taken using a confocal microscope Nikon Eclipse Ti using a 20x objective (for HtrA1 quantification) or a Zeiss LSM 880 Airyscan confocal microscope using a 20x or 63x objective (all other staining). Brightness was equally adjusted per antibody after image acquisition for display purposes only. For high magnification images of chondrocytes *in situ*, a z-stack was obtained, and a Z project using Maximum intensity was generated. Pictures were acquired with a resolution of 1024 × 1024 or above and 16-Bit.

### HtrA1^+^ cells quantification

Confocal images were obtained from each sample (undamaged and damaged areas) from each patient, resulting in a total of N = 52 images for OA-only and N = 43 images for DDH-OA (details in Supplementary Tables 1 and 2). Images were analyzed in ImageJ. To subtract background fluorescence before cell counting, the threshold was determined per each image using the corresponding negative controls where the primary antibody was omitted. Cell counting was performed in thresholded images using DAPI nuclear counterstain as reference for identifying cells. To account for differences in cellularity between samples, we calculated the percentage of HtrA1^+^ cells rather than taking fluorescence intensity. Total cells per image were counted using DAPI staining. The percentage of HtrA1^+^ cells calculated. Counts were done by two blind investigators. Mean values of % HtrA1^+^ cells and MMS were used for statistical analysis.

### Chondrocyte isolation and gene expression analysis

Minced cartilage from undamaged and damaged areas of the hips was incubated for 30 min in 0.2% Pronase (Roche) followed by 17 hours of incubation in 0.2% Collagenase D (Roche) prepared in high glucose DMEM with sodium pyruvate and glutamate (Sigma) + 10% heat-inactivated Fetal bovine serum (Sigma) + 1x Antibiotic-Antimycotic (Gibco) + 50 µg/ml ascorbic acid. Cells were passed through a 100 µm strainer, washed with PBS, counted and used for RNA isolation with Qiazol (Qiagen), following manufacturer’s instruction. Ratio 260/230 and 260/280 was obtained with NanoDrop One. 500 ng of total RNA were used for cDNA synthesis with iScript cDNA synthesis kit (Bio-Rad). One µl of this reaction was used for qPCR with SsoFast Eva Green qPCR kit (Bio-Rad). For gene expression analysis, we calculated 2^(−ΔCt)^ using 18 s as housekeeping gene. Human primers for HtrA1, 18 s, and IL-1β were from QuantiTect Primer assay (Qiagen). Results are shown as relative quantity (RQ) to 18 s.

### Statistical analysis

Local regression along with the Loess nonparametric method of fitting was used in estimating the local weighted regression surface (via weighted least squares) for the relationship between the mean HtrA1^+^ cells (%) and cartilage degeneration (mean MMS) in the OA-only images (total N = 52 obtained from 13 patients) and, separately, in the DDH-OA images (total N = 43 obtained from 9 patients). Specifically, this Loess fitting was based on local quadratic regression with a weighting kernel that was tricubic within a smoothing parameter span of 1.0 (for the OA-only sample) and 0.872 for the DDH-OA sample (100% and 87.2% around the point of prediction, respectively). The Loess curves for OA-only and DDH-OA resulted in different empirical patterns, therefore, Spearman correlation coefficient (*r*_*s*_) was estimated based on the empirical patterns and not on the pathological condition of the sample. The inter-rater reliability (using the ICC) between the two raters of MMS on the same OA-only samples was 0.910 (95% CI: 0.826 to 0.951) and on the same DDH-OA samples was 0.929 (95% CI: 0.871 to 0.962). The inter-rater reliability (using the ICC) between the two raters’ HtrA1^+^ counts on the same OA-only samples was 0.860 (95% CI: 0.749 to 0.921) and on the same DDH-OA samples was 0.915 (95% CI: 0.842 to 0.954). We used the mean of the two raters’ HtrA1^+^ counts and MMS in the analysis. Analyses were carried out using SAS software, version 9.4 (SAS Institute, Inc., Cary, NC, USA). The level of significance was set at α = 0.05 (two-tailed).

Finally, to compare % of HtrA1^+^ cells in undamaged OA-only versus damaged OA-only, we performed a paired Student’s t-test, with level of significance set as α = 0.05 (two-tailed). To compare % of HtrA1^+^ cells in undamaged OA-only versus undamaged DDH-OA, we performed an unpaired Student’s t-test, with level of significance set as α = 0.05 (two-tailed). For the relationship between HtrA1^+^ cells with age of patient, and cellularity of tissue, we performed Pearson’s correlation analysis. To compare for differences in gene expression of IL1-β and HtrA1 in the four groups, we performed a one-way ANOVA with Fisher’s LSD as post-hoc. Level of significance was set as α = 0.05. These analyses were done in GraphPad Prism 8.

## Results

### Relationship between HtrA1 and cartilage damage in OA-only samples

**Figure 1:**
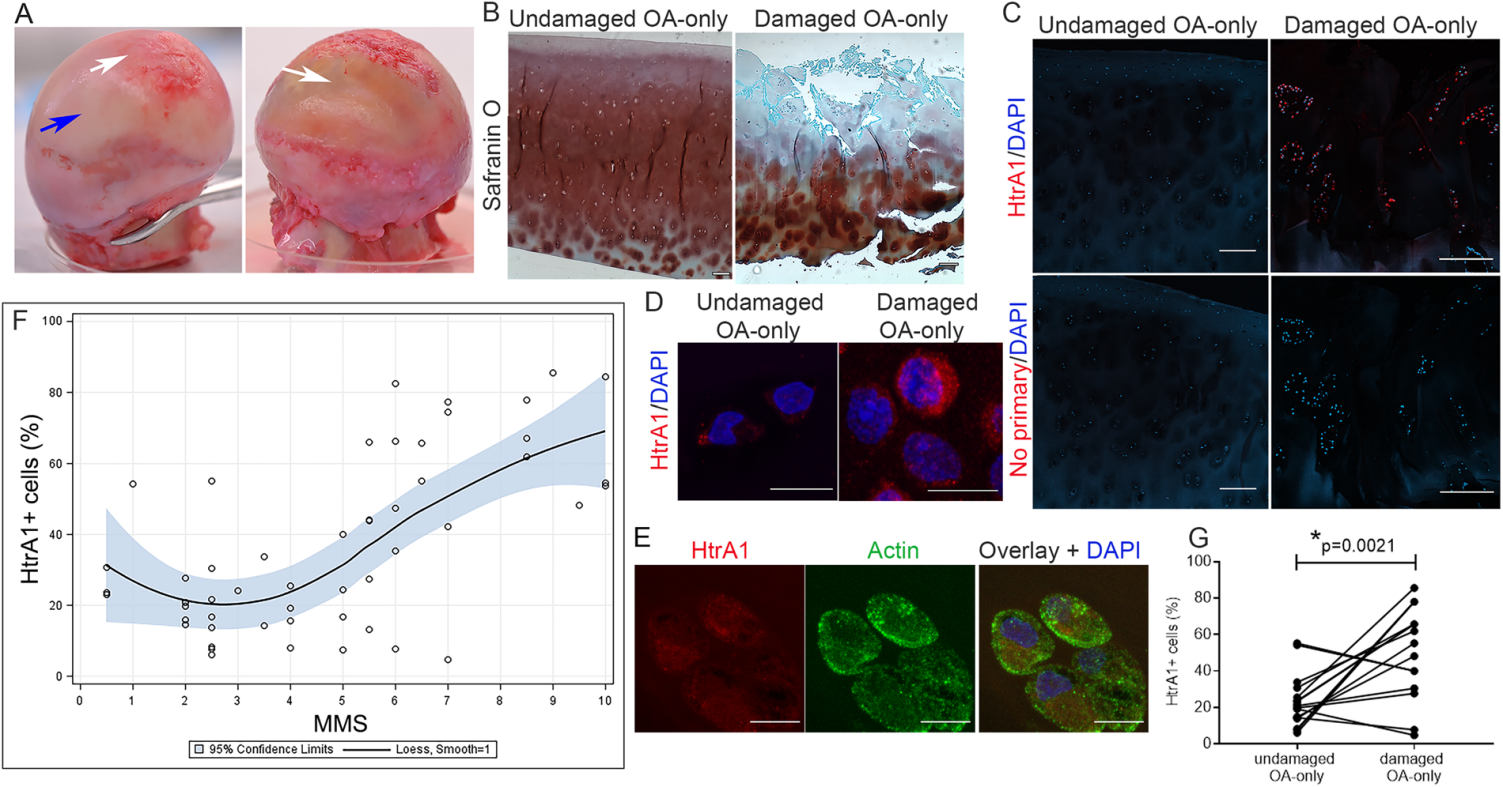
Percentage of HtrA1^+^ cells increases with degeneration in OA-only samples. (**A**) Representative images of femoral heads indicating undamaged areas (blue arrow) and damaged areas (white arrows). (**B**) Proteoglycan visualized with Safranin-O staining of undamaged area with MMS of 2.5 and a damaged area with MMS of 10. Scale bar is 200 µm. (**C**) HtrA1 immunostaining (red) showing HtrA1^+^ cells in a sample with MMS of 2.5 (undamaged) compared to a sample with MMS of 10 (damaged). DAPI was used as nuclear counterstain (blue). Lower panel shows negative controls where primary antibody was omitted, DAPI was used as nuclear staining. Scale bars in C are 200 µm. (**D**) Higher magnification image showing HtrA1 in red and nucleus in blue from the same undamaged and damaged samples from (**C**). Scale bars in D are 10 µm. (**E**) Higher magnification of HtrA1 (red) co-stained with Actin (green) and DAPI (nucleus, blue) showing intracellular HtrA1. Scale bars in E are 10 µm. (**F**) Local weighted regression with Loess fitting for the relationship between % HtrA1^+^ cells and MMS. Results shown as mean values with 95% confidence limits for the regression surface (blue area). N = total 52 images taken from 13 patients. (**G**) Paired comparison of % HtrA1^+^ cells between undamaged and damaged cartilage from OA-only samples. *Indicates a significant difference in a paired Student’s t-test with p = 0.0021. Data shown as mean of 2 blind counts. N = 16 pairs of images from 8 patients.

We collected samples from areas with no visible fibrillation and cartilage from areas with fibrillation in the OA-only patient group (Fig. 1A). From this group we obtained only undamaged cartilage from 2 patients, only damaged cartilage from 4 patients and both undamaged and damaged cartilage from 7 patients (Supplementary Table 1).

By first analyzing these samples as a patient independent pool, we were able to cover a range of OA severity, estimated by tissue damage using proteoglycan staining with Safranin O, and a MMS score (Fig. 1B). HtrA1 immunostaining appeared mostly located at the superficial and middle layers of cartilage (Fig. 1C). There was almost no detectable staining in the deeper zone (Supplementary Fig. S1). By using co-staining with actin, we determined that HtrA1 was mostly intracellular (Fig. 1D,E).

We performed a local weighted regression with Loess fitting to identify the relationship between the percentage of HtrA1^+^ cells and the MMS. The empirical pattern obtained by Loess curve revealed a relatively flat relationship with MMS from 0 to 3, but it was followed by an increasing positive linear relationship when MMS were greater than 3 (Fig. 1F). Therefore, we calculated conditional correlation coefficients for the groups with MMS from 0 to 3, and then when they were 3.5 to 10. The estimated Spearman correlation coefficient was *r*_*s*_ = −0.3076 (p = 0.2143) for scores between 0–3 (N = 18 images, from 7 patients) and *r*_*s*_ = 0.6589 (p < 0.0001) for MMS between 3.5–10 (N = 34 images, from 12 patients), demonstrating a low percentage of HtrA1^+^ cells in cartilage with no histological manifestation of OA and a high percentage in damaged cartilage. These results demonstrate that greater cartilage degeneration during the development of OA, as measured by MMS > 3, is related to a greater percentage of HtrA1^+^ cells, while in tissues with no histological manifestation of OA, the expression of HtrA1^+^ is low. Similarly, when cartilage images were analyzed as patient-dependent, we observed a lower percentage of HtrA1^+^ cells in undamaged cartilage compared to damaged cartilage from the same patient (p = 0.0021, Student’s t-test) (Fig. 1G).

**Figure 2:**
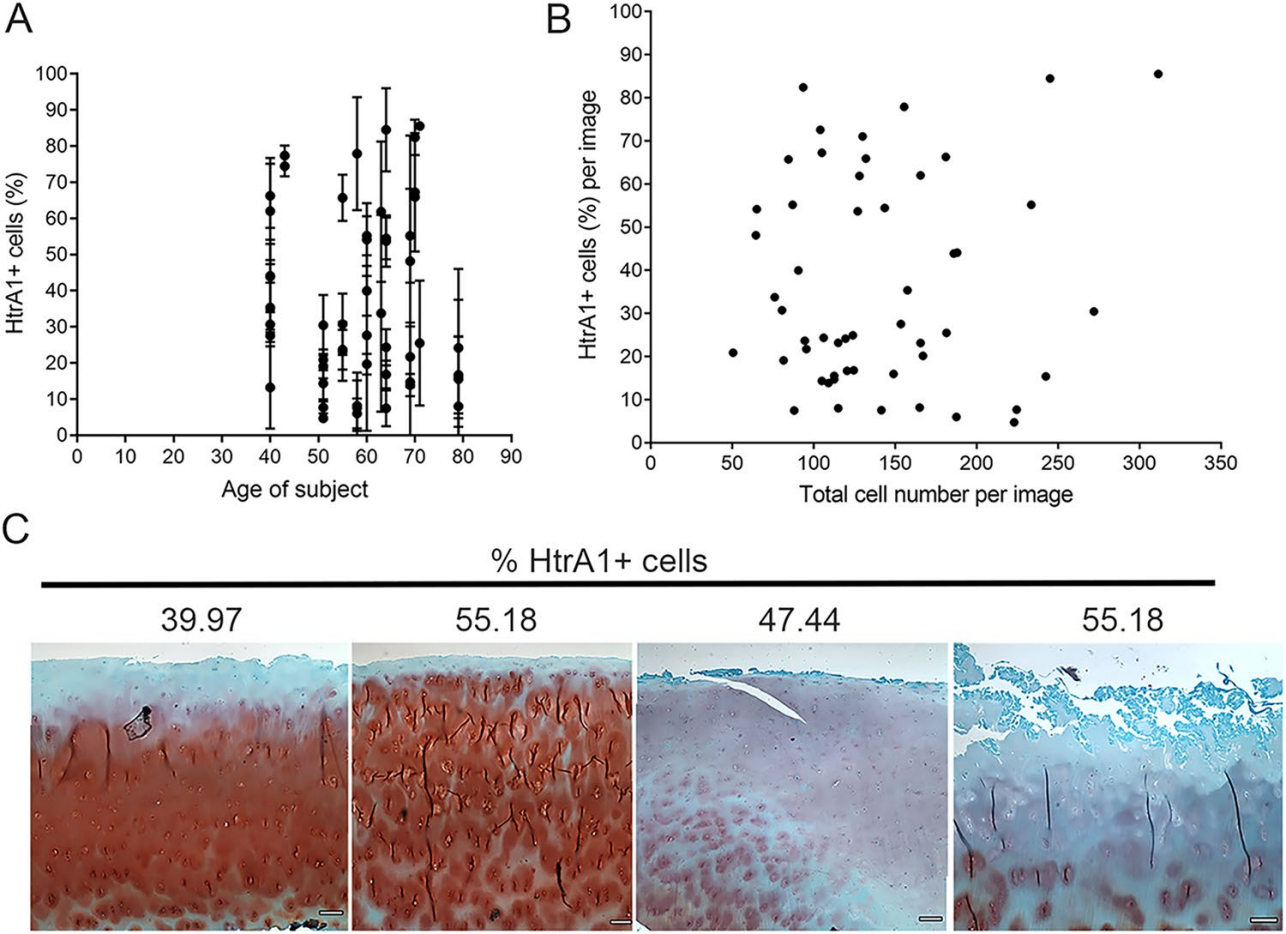
No relationship between the percentage of HtrA1^+^ cells and the age of patient, cellularity, or proteoglycan content. (**A**) Graph showing no apparent relationship between % HtrA1^+^ cells and age of patients. Results presented as mean per patient per condition (undamaged and damaged) with SD. N = 52 images from 13 patients. (**B**) Graph showing no relationship between % HtrA1^+^ cells and cellularity of tissue measured as total cell number per image. Results presented as mean of 2 blind counts. Error bars were omitted for clarity. N = 52 images from 13 patients. (**C**) Representative images of OA-only samples stained for Proteoglycan content with Safranin-O. Percentage of HtrA1^+^ cells shown. Scale bars are 200 µm.

We next analyzed whether the percentage of HtrA1^+^ cells depended on factors such as age, cellularity of tissue, and proteoglycan content. We did not find any apparent relationship with any of these factors in this study (Fig. 2A,B). While proteoglycan staining was inversely related to the MMS, we found that in samples with comparable percentage of HtrA1^+^ cells, the proteoglycan content varied widely (Fig. 2C).

### Relationship between MMP13 staining, cell death and loss of aggrecan and collagen II and cartilage damage in OA-only samples

In order to characterize, more specifically, the nature of any changes in the cartilage obtained, we investigated the distribution of the two main cartilage ECM macromolecules, collagen II and aggrecan (protein component of proteoglycans), and chondrocyte cell death, using IHC.

**Figure 3:**
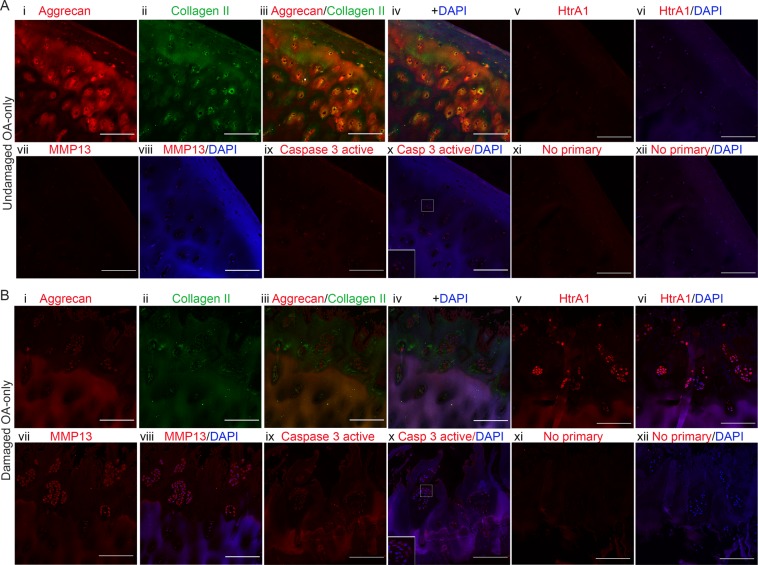
Loss of ECM proteins and increase in proteases staining in damaged OA-only samples. (**A**) Panel showing an undamaged OA-only sample with MMS 2.5 stained for Aggrecan (i, red), Collagen II (ii, green), overlay (iii), overlay with DAPI (iv), HtrA1 (v, red), HtrA1 + DAPI (vi), MMP13 (vii, red), MMP13 + DAPI (viii), Caspase-3 active (ix, red), Caspase-3 active + DAPI (x) with inset for cells in higher magnification, No primary control (xi, red), and No primary control + DAPI (xii). Only Aggrecan and Collagen II were co-stained on the same sample. The images for the other antibodies’ staining are in equivalent areas. (**B**) Same array of panels for a damaged OA-only sample with MMS 10. Scale bars are 200 µm. Images were taken with 20x objective.

Aggrecan and collagen II staining was strong in undamaged cartilage, but less in damaged OA-only cartilage (Fig. 3A,B, panel i-iv). In contrast, both HtrA1 (Fig. 3A,B, panel v-vi) and MMP13 (Fig. 3A,B, panel vii-viii) showed a reverse pattern of staining (high in damaged cartilage). To investigate chondrocyte death IHC of the cleaved (active) form of caspase-3 was performed. The results showed that there was an increase in caspase-3 active staining in damaged OA-only samples compared to undamaged cartilage (Fig. 3A,B, panel ix-x). The staining of deeper areas of cartilage are shown in Supplementary Fig. S1.

### Relationship between HtrA1 and cartilage damage in DDH-OA samples

**Figure 4:**
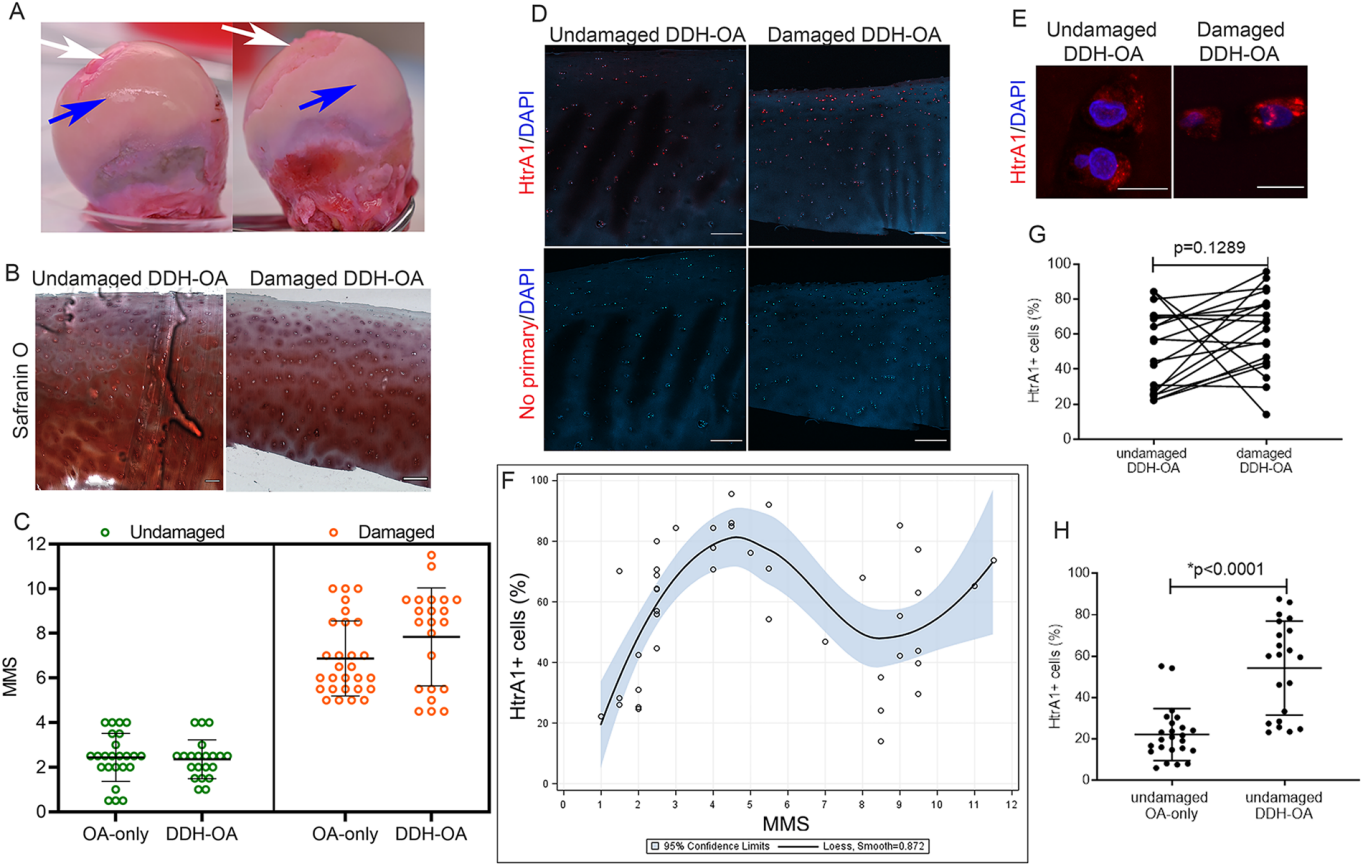
Increase in percentage of HtrA1^+^ cells observed in undamaged DDH-OA samples. (**A**) Representative images of femoral heads indicating undamaged areas (blue arrow) and damaged areas (white arrows). (**B**) Safranin-O staining of an undamaged DDH-OA sample with MMS of 2.5 and a damaged DDH-OA samples with MMS of 4.5. (**C**) Graph showing comparison between MMS of OA-only versus DDH-OA for the undamaged (green symbols) and the damaged (orange symbols) groups. Data shown as mean ± SD of two independent raters. (**D**) Representative confocal images of HtrA1 staining to show HtrA1^+^ cells in undamaged versus damaged DDH-OA cartilage. DAPI was used as a nuclear counterstain. Lower panel is negative control where the primary antibody was omitted. Scale bars are 200 µm. (**E**) Higher magnification images of HtrA1 in chondrocytes *in situ* in undamaged and damaged DDH-OA cartilage. Scale bars are 10 µm. (**F**) Local weighted regression with Loess fitting for the relationship between % HtrA1^+^ cells and MMS. Results shown as mean values with 95% confidence limits for the regression surface (blue area). N = 43 images from 9 patients. (**G**) Graph of paired observations showing no significant difference between % of HtrA1^+^ cells in undamaged versus damaged DDH-OA cartilage. Student’s t-test p = 0.1289 (two-tailed). Results shown as mean of 2 blind counts. N = 19 pairs of images from 7 patients. (**H**) Unpaired comparison of % HtrA1^+^ cells between undamaged OA-only and undamaged DDH-OA cartilage samples. *Indicates significant difference in unpaired Student’s t-test with p < 0.0001. Results shown as mean of 2 blind counts with SD. N = 24 images from 9 patients for undamaged OA-only; N = 21 images from 7 patients for damaged DDH-OA.

We collected DDH-OA cartilage from undamaged and damaged areas (Fig. 4A,B). From this group we obtained only damaged cartilage from 2 patients and both undamaged and damaged cartilage from 7 patients (Supplementary Table 2). A graph comparing MMS for undamaged and damaged DDH-OA with OA-only is shown in Fig. 4C. Summarized statistics of MMS for both groups are shown in Supplementary Table 3 and 4. Tissue immunofluorescence of DDH-OA cartilage indicated that HtrA1 staining was increased in the surface and middle layers of the cartilage, similar to that seen in OA-only samples (Fig. 4D and Supplementary Fig. S1), primarily intracellular staining (Fig. 4E).

The local weighted regression with Loess fitting revealed a cubic relationship between the percentage of HtrA1^+^ cells and MMS in DDH-OA samples (Fig. 4F). Specifically, the empirical pattern of the Loess curve for DDH-OA showed a growing positive relationship between the percentage of HtrA1^+^ cells and MMS from 0 to 4.5, different from OA-only cartilage where the positive relationship was observed between MMS 3.5 to 10 (Fig. 4F). Therefore, we calculated the conditional correlation in the group with MMS from 0 to 4.5, and found an estimated Spearman correlation coefficient of *r*_*s*_ = 0.8856 (p < 0.0001) (N = 24 images, from 7 patients).

When we analyzed images as patient-dependent, we found that the percentage of HtrA1^+^ cells in undamaged DDH-OA was similar to damaged DDH-OA samples (Fig. 4G. Student’s t-test, p = 0.1289), indicating that HtrA1 upregulation was present even in less damaged cartilage in DDH-OA preceding histological changes. The percentage of HtrA1^+^ cells was significantly higher in undamaged DDH-OA than in undamaged OA-only (Fig. 4H, Student’s t-test, p < 0.0001).

RNA obtained from OA-only and DDH groups were analyzed for HtrA1 gene expression. Results show no significant differences between any of the groups (Supplementary Fig. S2A). HtrA1 mRNA did not correlate with the percentage of HtrA1^+^ cells (Supplementary Fig. S2B). Similar to what was observed for OA-only samples, we did not find any relationship between the percentage of HtrA1^+^ cells with age, cellularity, and proteoglycan content (Supplementary Fig. 2C–E).

### Relationship between MMP13 staining, cell death and loss of aggrecan and collagen II and cartilage damage in DDH-OA samples

**Figure 5:**
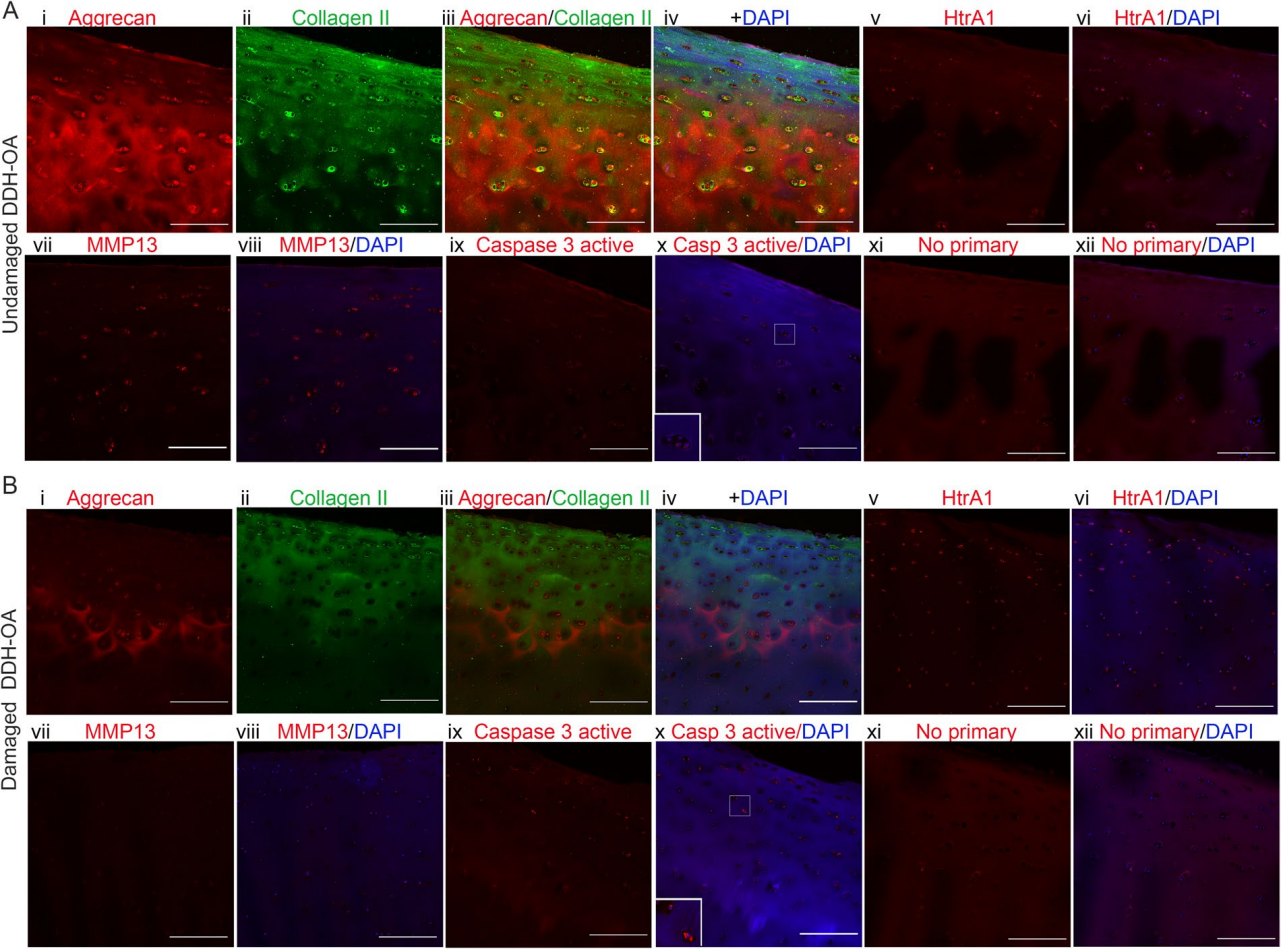
Signs of remodeling but not of significant ECM degradation were detected in undamaged DDH-OA samples. (**A**) Panel showing an undamaged DDH-OA sample with MMS 2.5 stained for Aggrecan (i, red), Collagen II (ii, green), overlay (iii), overlay with DAPI (iv), HtrA1 (v, red), HtrA1 + DAPI (vi), MMP13 (vii, red), MMP13 + DAPI (viii), Caspase-3 active (ix, red), Caspase-3 active + DAPI (x) with inset for cells in higher magnification, No primary control (xi, red), and No primary control + DAPI (xii). Only Aggrecan and Collagen II were co-stained on the same sample. The images for the other antibodies’ staining are in equivalent areas. (**B**) Same array of panels for a damaged DDH-OA sample with MMS 4.5. Scale bars are 200 µm. Images were taken with 20x objective.

Since chondrocytes in undamaged DDH-OA cartilage resembled damaged OA-only in the matrix protease HtrA1 expression, we investigated aggrecan and collagen II IHC. There was positive staining for both aggrecan and collagen II in undamaged DDH-OA cartilage. As expected with cartilage degeneration, the staining for both proteins was less in damaged DDH-OA cartilage (Fig. 5A,B, panel i-iv). HtrA1^+^ cells were detected in both undamaged and damaged DDH-OA samples (Fig. 5A,B, panel v-vi). MMP13 staining in undamaged DDH-OA was greater compared to undamaged OA-only; however, MMP13 staining in damaged DDH-OA cartilage was low (Fig. 5A,B, panel vii-viii). The analysis of cell death by staining for cleaved caspase-3 active revealed the presence of few apoptotic cells in undamaged DDH-OA cartilage (Fig. 5A,B, panel ix-x). Staining of all these antibodies in deeper areas of cartilage are shown in Supplementary Fig. S3).

### Signs of chondrocyte mechanical stress present in OA cartilage were observed in undamaged DDH-OA cartilage

**Figure 6:**
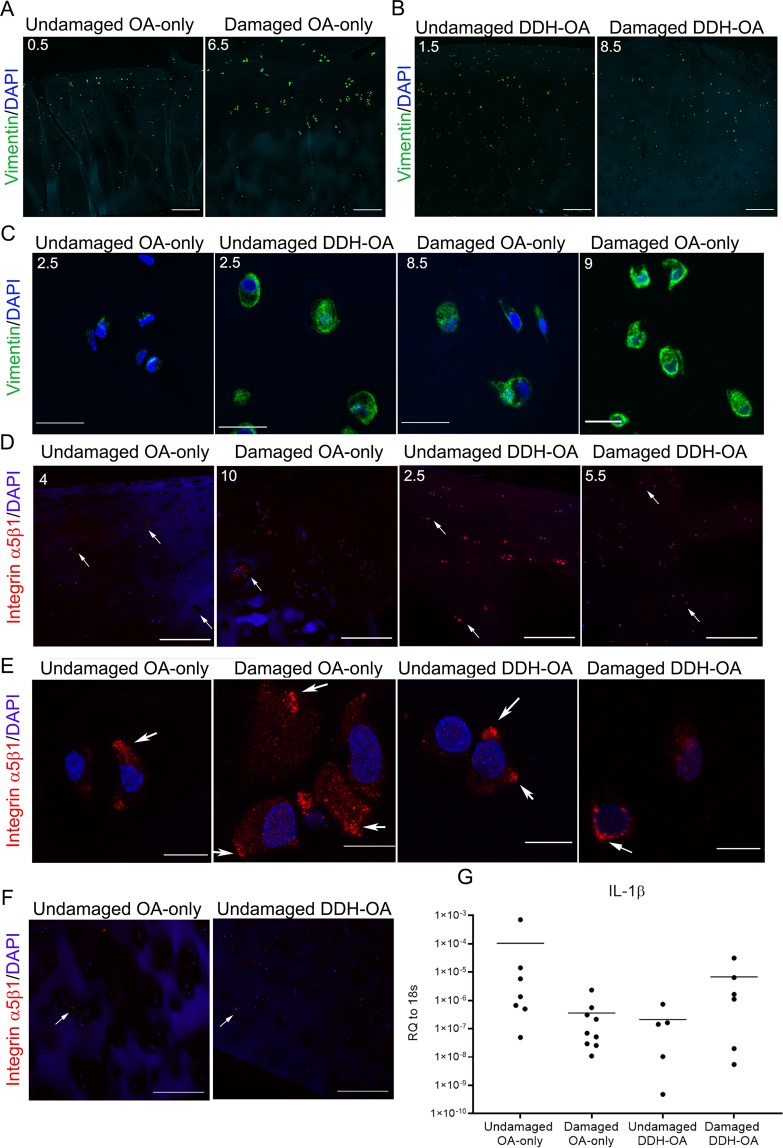
Additional signs of osteoarthritic pathological phenotype of chondrocytes were detected in undamaged DDH-OA samples. (**A**) Vimentin intermediate filaments staining (green) of an undamaged and a damaged OA-only sample. DAPI was used as nuclear counterstain. (**B**) Vimentin (green) and DAPI staining of undamaged and damaged DDH-OA cartilage. (**C**) Images of vimentin + DAPI staining in chondrocytes *in situ* showing that undamaged DDH-OA samples have more vimentin staining than undamaged OA-only with the same MMS and is more comparable to samples of damaged OA-only with higher MMS. MMS are indicated in the upper left corner of each picture. (**D**) Integrin α_5_β_1_ (red) + DAPI staining of all groups. Surface and middle layers are shown. White arrows indicate examples of positive cells throughout cartilage depth. (**E**) Images of chondrocytes *in situ* from samples in **D**. White arrows indicate integrin α_5_β_1_ clustering. (**F**) Integrin α_5_β_1_ staining in deep layers of undamaged OA-only and undamaged DDH-OA cartilage. White arrows indicate examples of positive cells. (**G**) mRNA expression of IL-1β in the 4 groups of samples. No significant differences were detected between the groups. Values are shown as relative quantity (RQ) to 18 s in Log_10_ scale for better display. Lines represent average. Scale bars for **A, B, D**, and **F** are 200 µm. Scale bars for C and E are 10 µm.

Since there was evidence of ECM remodeling, but a lack of significant histological matrix degeneration in undamaged DDH-OA cartilage, we investigated whether there were any changes in the cytoskeleton in chondrocytes in undamaged DDH-OA cartilage. Vimentin intermediate filaments are critical for chondrocyte structural mechanics and accumulate under higher mechanical stress. IHC for vimentin revealed stronger fluorescence intensity in chondrocytes from the surface and middle layers in damaged OA-only cartilage when compared to chondrocytes from undamaged cartilage from the same patient (Fig. 6A). Similar to that seen for HtrA1 IHC, higher levels of vimentin staining was observed in undamaged DDH-OA cartilage (Fig. 6B).

Analysis of higher magnification images revealed thicker vimentin intermediate filament network in chondrocytes from undamaged DDH-OA cartilage resembling more those in damaged OA-only with higher MMS, than undamaged OA-only (Fig. 6C).

Qualitative analysis of confocal images of the mechanosensor protein, integrin α_5_β_1_, showed that although staining was present in undamaged OA-only samples and more in damaged OA-only, we observed that both undamaged and damaged DDH-OA cartilage had cells positive for this integrin more frequently found throughout the tissue (Fig. 6D). In all samples, the distribution of integrin α5β1 was spread throughout the cartilage depth (Fig. 6D). Integrin α_5_β_1_ staining had some clustering at the cell periphery in all samples (Fig. 6E).

### Gene expression analysis in OA-DDH versus OA-only cartilage

To seek whether activation of an inflammatory pathway in DDH-OA chondrocytes could have upregulated HtrA1 and MMP13 staining observed in this study, we investigated the mRNA expression of the pro-inflammatory cytokine IL-1β. The comparison between the four study-groups did not reveal statistical significance between the groups (Fig. 6G).

## Discussion

In this study, we show evidence that changes are seen at the chondrocyte level in hip cartilage from patients with DDH, a disorder that leads to abnormal forces in the hip, before detectable changes in structure of the cartilage. Using aggrecan and collagen II IHC staining (in addition to standard histological techniques) we have shown that undamaged DDH cartilage has minimal ECM changes but that an upregulation of the serine protease HtrA1, reorganization of vimentin intermediate filaments that influence chondrocyte mechanical stability, and more staining of cells with clusters of the mechanosensor integrin α_5_β_1_ is detected. These findings indicate that changes in chondrocytes precede ECM degeneration in DDH, leading us to propose that undamaged cartilage obtained from DDH patients may be considered as a model to study early OA changes.

Hips from DDH patients have an increase in medially directed forces, resulting in altered joint reaction forces^32^, with focal increases in stress at a primary point of contact between the femoral head and the acetabulum^6^ in comparison to more evenly distributed forces seen in the normal hip^33^. Contrary to what we observed for undamaged OA-only cartilage, undamaged DDH-OA cartilage had a higher percentage of HtrA1^+^ cells, even though there was no apparent surface fibrillation or proteoglycan loss. This observation was the first indication that in undamaged DDH-OA cartilage, chondrocytes are responding to a mechanical stresses prior to detectable histological manifestations.

In this study, HtrA1 has been investigated because of its role regulating the TGFβ pathway and cleaving fibronectin^16,34^, two events that influence cartilage pathophysiology^17,35^ and HtrA1 has been associated with OA in a number of reports^16,19–23^, including evidence for early upregulation during cartilage degeneration^21^. We first investigated changes in HtrA1 protein expression *in situ*, in cartilage samples with a range of cartilage degeneration degree. In normal cartilage, HtrA1 protein is located intracellularly, with most detected in surface and middles zone chondrocytes, as reported previously in knee and temporomandibular cartilage in animal models^20,21^ and human hip cartilage^19^. In our study, HtrA1 first appeared in cartilage with minor damage, indicating that HtrA1 expression is an early event in the progression of OA, which has been previously suggested^21^. We did not find a relationship between HtrA1 gene expression and the percentage of HtrA1^+^ cells; therefore, we propose that changes in HtrA1 are likely due to a decrease in protein degradation rather than synthesis *de novo*. Factors that could affect HtrA1 expression are age, tissue cellularity, and proteoglycan content. Previous studies have linked HtrA1 with an inhibition of proliferation^36^; however, we did not find any relationship with cellularity in this study. Nor did we find an association of intracellular HtrA1 with age or with proteoglycan content in the cartilage. It has been shown that HtrA1 cleavage of aggrecan generates a new epitope and that this epitope is more abundant in knee cartilage from OA patients, indicating that HtrA1 may have a crucial role in proteoglycan integrity in OA^19^. A limitation to our study is that we did not account for any exported HtrA1, so we can only conclude that the loss in proteoglycan *per se* is not related to the increase in the intracellular subpopulation of HtrA1. Another limitation is our small number of subjects. Sex is also a potential factor that may affect HtrA1— although this relationship has not been previously established or reported. The current study did not evaluate a gender effect; however, evaluating sex as another potential salient factor for this protein will require a larger sample size and could be a focus of future research.

Vimentin intermediate filaments are essential components of the chondrocyte cytoskeleton and are upregulated in response to mechanical stress^9,10,37,38^, up to tissue damaging stress where vimentin networks can break and disassemble^39^. Both OA and DDH-OA cartilage experience abnormal loading^32,40^. When cartilage suffers injury, the extracellular matrix components such as proteoglycan and collagen fibers lose the arrangement that allows them to withhold water and to regulate hydrostatic pressure^40,41^ affecting chondrocyte load. Deformation resulting from compression is higher at the surface and middle layers of cartilage^42^, and since the mechanical properties of ECM are altered in OA, mechanical loading of chondrocytes can be exacerbated. To evaluate whether the structural mechanics of chondrocytes was affected by the abnormal mechanics experienced in DDH-OA cartilage, we investigated changes in vimentin filaments arrangement. Our results revealed that vimentin filaments organized into thicker networks as cartilage degradation advanced, with the most robust staining predominantly located at the surface and middle layers. This pattern, similar to HtrA1 staining, was located in areas that are predicted to be the most deformed under compression forces^42^. Interestingly, the same reorganization of vimentin filaments was detected in undamaged DDH-OA cartilage, indicating a cellular response to a common stimulation in both damaged OA cartilage and undamaged DDH-OA cartilage.

To further study cellular responses to mechanical changes in cartilage, we evaluated integrin α_5_β_1_. This integrin is the primary receptor for fibronectin and can also function as a mechanoreceptor^43,44^. During the progression of OA, chondrocytes experience changes in integrin expression^45–47^. Since integrin α_5_β_1_ binds not only regular fibronectin but also fibronectin fragments, depending on the ongoing physiological process, its activation can result in a detrimental physiological response^18,48^ or a positive one, promoting cell survival^49^. Although there are reports of the upregulation of integrin α_5_β_1_ in OA^46^, there is a lack of a detailed analysis of integrin clustering in chondrocytes *in situ* as a response to the progression of OA. Here we found that integrin α_5_β_1_ forms small clusters in chondrocytes *in situ* in OA cartilage and in DDH-OA cartilage, and that these clusters are more frequently found in undamaged DDH-OA cartilage.

The activation of inflammatory cascades is one of the factors that may contribute to cartilage degeneration, although there is no evidence that inflammation is related to DDH. Since upregulation of MMP13 can occur as response to pro-inflammatory cytokines such as IL-1β^50^, we investigated whether DDH-OA cartilage had higher expression of this cytokine. Interestingly, we did not find significant differences in mRNA expression of IL-1β between the four groups studied here, however, it is important to acknowledge that our sample number is low. Therefore, we can only conclude that for the samples analyzed in this study, chondrocytes do not upregulate IL-1β in DDH-OA.

Since DDH hips are exposed to abnormal mechanical load, we propose that the thickening of vimentin filaments and the accumulation of integrin α_5_β_1_ at the cell periphery represents a response to changes in mechanical stimulation prior to histological changes in the cartilage. Tissue structural factors can affect the cell’s perception of mechanical stimulation and its mechanotransduction^51^. Studies on biophysical characterization of the ECM in OA cartilage have shown that collagen fibers undergo stiffening, due to increased fiber cross-linking directly affecting the elasticity of the ECM^52,53^. This higher matrix stiffness translates into activation of the Rho-ROCK-MLC pathway and increased formation of focal adhesions^54^. Higher matrix stiffness also affects polymerization of vimentin filaments, leading to a thicker network^55^. Moreover, it has been shown that vimentin stabilizes focal adhesions when endothelial cells are exposed to shear stress^56^ and that fibroblasts from vimentin-null mice have more irregular and less mechanically stable focal contacts^57^ (reviewed in^58^). This evidence, together with the lack of IL-1β upregulation in chondrocytes, suggests that the histological changes that we observed for both vimentin and integrin α_5_β_1_ are likely due to alterations at the mechanical environment.

Interestingly, MMP13 not only increases with IL-1β, but there is evidence that mechanical induction is also involved in its upregulation^59,60^, and in fact MMP13 is upregulated early in a rat model for DDH^29^, with a similar staining pattern to the one we observed. We detected more MMP13 in undamaged DDH-OA compared to undamaged OA-only, and both samples had similar aggrecan and collagen II staining, indicating that the increase in MMP13 is an early event, like that observed in a rat model for DDH^29^. We propose that there is an early induction of matrix turnover by MMP13 and possibly HtrA1. This supports the hypothesis of microstructural changes in the immediate chondrocyte niche that affect their internal microstructure.

The results in this study suggest that physiological changes occur early in chondrocytes, before histological manifestations of OA. We propose that these changes are a response to an altered chondrocyte microenvironment. Moreover, our lack of evidence for inflammatory involvement leads us to further propose that mechanical changes are likely to be responsible for the pathological phenotype observed in chondrocytes. Although more research is required to empirically test this hypothesis, this DDH cartilage environment provides a novel opportunity to study early events of OA and provide a possible opportunity for the developments of therapeutic targets for OA.

## Supplementary information

Supplementary Material
