# Supplementary material for: Early-Onset Osteoarthritis originates at the chondrocyte level in Hip Dysplasia
Source: Sci Rep. 2020 Jan 17;10:627. doi: 10.1038/s41598-020-57431-x (PMC6969105; doi:10.1038/s41598-020-57431-x)
Supplement: Supplementary file 1 — Supplementary Material [file 41598_2020_57431_MOESM1_ESM.pdf]

Supplementary Material

Early-Onset Osteoarthritis originates at the chondrocyte level in Hip Dysplasia.

Paula A. Hernandez[1*], Joel Wells[1], Emiliya Usheva[1], Paul Nakonezny[2], Zahra Barati[1], Roberto Gonzalez[1], Layla Kassem[1] and Frances M.D. Henson[3]

[1] Department of Orthopaedic Surgery, University of Texas Southwestern Medical Center, Dallas, TX 75390, USA
[2]Department of Population and Data Sciences, Division of Biostatistics, University of Texas Southwestern Medical Center, Dallas, TX 75390, USA
[3] Division of Trauma and Orthopaedic Surgery, University of Cambridge, Cambridge, CB2 2QQ, UK
*** Paula.Hernandez2@UTSouthwestern.edu**

Supplementary Table 1
Table 2
Table 3
Table 4

Supplementary Fig. S1
Fig. S2
Fig. S3

Supplementary Table 1

Patient #	Age/sex	Condition	MMS person 1	MMS person 2	average MMS	HtrA+ cells/total cells person 2	% HtrA count person 2	HtrA+ cells/total cells person 3	% HtrA count person 3	average % HtrA1
1	79F	U	3	1	2	12/150	8.00	39/163	23.93	15.96
1	79F	U	4	2	3	12/138	8.70	40/101	39.60	24.15
1	79F	U	4	1	2.5	13/142	9.15	24/99	24.24	16.70
1	79F	U	5	3	4	0/122	0.00	32/103	31.07	15.53
1	79F	U	5	3	4	2/110	1.82	17/120	14.17	7.99
2	58F	U	4	1	2.5	6/196	3.06	16/179	8.94	6.00
2	58F	U	4	1	2.5	6/185	3.24	19/145	13.10	8.17
2	58F	U	4	1	2.5	1/152	0.66	19/131	14.50	7.58
2	58F	D	9	8	8.5	111/166	66.87	129/145	88.97	77.92
3	60M	U	2	2	2	28/129	21.71	21/119	17.65	19.68
3	60M	U	3	1	2	15/167	8.98	76/164	46.34	27.66
4	55F	U	0	1	0.5	35/121	28.93	19/109	17.43	23.18
4	55F	U	0	1	0.5	23/93	24.73	25/68	36.76	30.75
4	55F	U	0	1	0.5	27/98	27.55	18/91	19.78	23.67
4	55F	D	6	7	6.5	52/85	61.18	59/84	70.24	65.71
5	40M	D	6	5	5.5	13/250	5.20	50/235	21.28	13.24
5	40M	D	6	5	5.5	76/203	37.44	85/169	50.30	43.87
5	40M	D	6	5	5.5	108/211	51.18	61/165	36.97	44.08
5	40M	D	6	5	5.5	61/212	28.77	25/95	26.32	27.54
5	40M	D	7	7	7	29/161	18.01	115/173	66.47	42.24
5	40M	D	7	5	6	96/186	51.61	74/171	43.27	47.44
5	40M	D	7	5	6	132/182	72.53	108/180	60.00	66.26
5	40M	D	7	5	6	70/174	40.23	43/141	30.50	35.36
6	63M	U	4	3	3.5	44/83	53.01	10/69	14.49	33.75
6	63M	D	8	9	8.5	64/133	48.12	93/123	75.61	61.87
7	71F	U	5	3	4	46/122	37.70	32/241	13.28	25.49
7	71F	D	9	9	9	256/301	85.05	277/322	86.02	85.54
8	69F	U	3	1	2	4/130	3.08	25/95	26.32	14.70
8	69F	U	3	2	2.5	16/137	11.68	13/81	16.05	13.86
8	69F	U	3	2	2.5	17/108	15.74	23/83	27.71	21.73
8	69F	D	9	10	9.5	20/85	23.53	32/44	72.73	48.13

8	69F	D	6	7	6.5	114/248	45.97	141/219	64.38	55.18
9	70F	D	8	9	8.5	59/106	55.66	82/104	78.85	67.25
9	70F	D	6	6	6	75/95	78.95	79/92	85.87	82.41
9	70F	D	5	6	5.5	87/132	65.91	N/A	N/A	65.91
10	43F	D	6	8	7	79/109	72.48	81/106	76.42	74.45
10	43F	D	6	8	7	108/136	79.41	101/134	75.37	77.39
11	60M	U	2	3	2.5	59/100	59.00	38/74	51.35	55.18
11	60M	U	1	1	1	32/68	47.06	38/62	61.29	54.17
11	60M	D	4	6	5	33/94	35.11	39/87	44.83	39.97
12	51M	U	4	3	3.5	12/107	11.21	18/103	17.48	14.35
12	51M	U	5	3	4	14/84	16.67	17/79	21.52	19.09
12	51M	U	1	3	2	11/48	22.92	10/53	18.87	20.89
12	51M	D	6	6	6	18/203	8.87	16/246	6.50	7.69
12	51M	D	8	6	7	10/233	4.29	11/213	5.16	4.73
12	51M	U	2	3	2.5	88/242	36.36	74/302	24.50	30.43
13	64F	D	6	4	5	11/100	11.00	3/76	3.95	7.47
13	64F	D	6	4	5	25/128	19.53	17/121	14.05	16.79
13	64F	D	6	4	5	31/111	27.93	21/101	20.79	24.36
13	64F	D	10	10	10	227/245	92.65	187/245	76.33	84.49
13	64F	D	10	10	10	89/152	58.55	68/135	50.37	54.46
13	64F	D	10	10	10	84/143	58.74	54/111	48.65	53.69

Supplementary Table 1: Results obtained for OA-only samples. MMS was assessed by 2 blind investigators. The counts for HtrA1^{+} cells per field of view was done by 2 blind investigators and using confocal imaged taken with 10x objective. Averages of MMS and averages of % HtrA1^{+} cells were used for statistics. MMS of 4 and below were considered as undamaged (U), while the other samples were considered as damaged (D).

Supplementary Table 2

Patient #	Age/sex	Condition	MMS person 1	MMS person 2	average MMS	HtrA+ cells/total cells person 2	% HtrA count person 2	HtrA+ cells/total cells person 3	% HtrA count person 3	average % HtrA1
1	72F	D	12	11	11.5	39/54	72.22	30/40	75.00	73.61
1	72F	D	9	10	9.5	22/59	37.29	16/38	42.11	39.70
2	62M	D	9	9	9	89/99	89.90	62/77	80.52	85.21
2	62M	D	11	11	11	39/59	66.10	27/42	64.29	65.19
2	62M	D	6	5	5.5	74/106	69.81	70/97	72.16	70.99
3	41F	U	2	4	3	209/230	90.87	161/207	77.78	84.32
3	41F	U	3	5	4	150/171	87.72	112/138	81.16	84.44
3	41F	D	7	10	8.5	9/69	13.04	9/59	15.25	14.15
3	41F	D	7	10	8.5	17/69	24.64	31/68	45.59	35.11
4	62F	U	4	1	2.5	60/114	52.63	90/106	84.91	68.77
4	62F	U	2	1	1.5	61/122	50.00	101/112	90.18	70.09
4	62F	D	8	9	8.5	109/505	21.58	110/408	26.96	24.27
4	62F	D	4	6	5	72/94	76.60	53/70	75.71	76.16
5	67F	U	1	2	1.5	14/67	20.90	19/61	31.15	26.02
5	67F	U	1	3	2	41/82	50.00	25/72	34.72	42.36
5	67F	U	3	2	2.5	42/85	49.41	31/78	39.74	44.58
5	67F	D	6	5	5.5	102/112	91.07	93/100	93.00	92.04
5	67F	D	9	10	9.5	42/54	77.78	43/56	76.79	77.28
5	67F	D	9	9	9	20/38	52.63	25/43	58.14	55.39
6	48M	U	3	2	2.5	98/122	80.33	94/118	79.66	79.99
6	48M	U	3	2	2.5	66/100	66.00	62/99	62.63	64.31
6	48M	U	3	2	2.5	74/97	76.29	43/83	51.81	64.05
6	48M	D	4	5	4.5	134/145	92.41	111/139	79.86	86.13
6	48M	D	4	5	4.5	164/187	87.70	118/144	81.94	84.82
6	48M	D	4	5	4.5	175/185	94.59	145/150	96.67	95.63
7	23M	U	3	2	2.5	98/143	68.53	53/116	45.69	57.11
7	23M	U	3	2	2.5	70/111	63.06	42/86	48.84	55.95
7	23M	U	3	2	2.5	77/104	74.04	66/98	67.35	70.69
7	23M	D	4	7	5.5	221/346	63.87	146/325	44.92	54.40
7	23M	U	2	6	4	146/185	78.92	114/149	76.51	77.71
7	23M	U	2	6	4	126/152	82.89	81/138	58.70	70.80
8	52F	U	3	1	2	39/123	31.71	26/138	18.84	25.27
8	52F	U	3	1	2	36/101	35.64	25/95	26.32	30.98
8	52F	U	3	1	2	21/79	26.58	19/83	22.89	24.74
8	52F	D	10	9	9.5	78/146	53.42	60/175	34.29	43.86
8	52F	D	10	9	9.5	63/189	33.33	60/230	26.09	29.71
8	52F	D	10	9	9.5	81/116	69.83	60/107	56.07	62.95

9	37M	U	1	2	1.5	30/113	26.55	26/86	30.23	28.39
9	37M	U	1	1	1	31/128	24.22	22/109	20.18	22.20
9	37M	U	1	1	1	32/117	27.35	21/123	17.07	22.21
9	37M	D	8	8	8	51/75	68.00	42/62	67.74	67.87
9	37M	D	10	8	9	52/126	41.27	58/135	42.96	42.12
9	37M	D	6	8	7	54/99	54.55	37/95	38.95	46.75

Supplementary Table 2: Results obtained for DDH-OA samples. MMS was assessed by 2 blind investigators. The counts for HtrA1$^+$ cells per field of view was done by 2 blind investigators and using confocal imaged taken with 10x objective. Averages of MMS and averages of % HtrA1$^+$ cells were used for statistics. Samples with average MMS of 4 and below were considered as undamaged (U), while the other samples were considered as damaged (D).

Supplementary Table 3

Variable	N	Mean	Std Dev	Median	Quartile Range	25th Pctl	75th Pctl	Range	Minimum	Maximum
MMS_rater1	52	5.135	2.582	5.500	3.000	3.500	6.500	10.000	0	10.000
MMS_rater2	52	4.519	2.907	4.000	4.500	2.000	6.500	9.000	1.000	10.000
MMS_avg	52	4.827	2.647	5.000	4.000	2.500	6.500	9.500	0.500	10.000

Supplementary Table 3: Summary statistics for the MMS values obtained by two raters for the OA-only group.

Supplementary Table 4

Variable	N	Mean	Std Dev	Median	Quartile Range	25th Pctl	75th Pctl	Range	Minimum	Maximum
MMS_rater1	43	5.047	3.251	4.000	5.000	3.000	8.000	11.000	1.000	12.000
MMS_rater2	43	5.279	3.439	5.000	7.000	2.000	9.000	10.000	1.000	11.000
MMS_avg	43	5.163	3.234	4.500	6.000	2.500	8.500	10.500	1.000	11.500

Supplementary Table 4: Summary statistics for the MMS values obtained by two raters for the DDH-OA group.

Supplementary Fig. S1

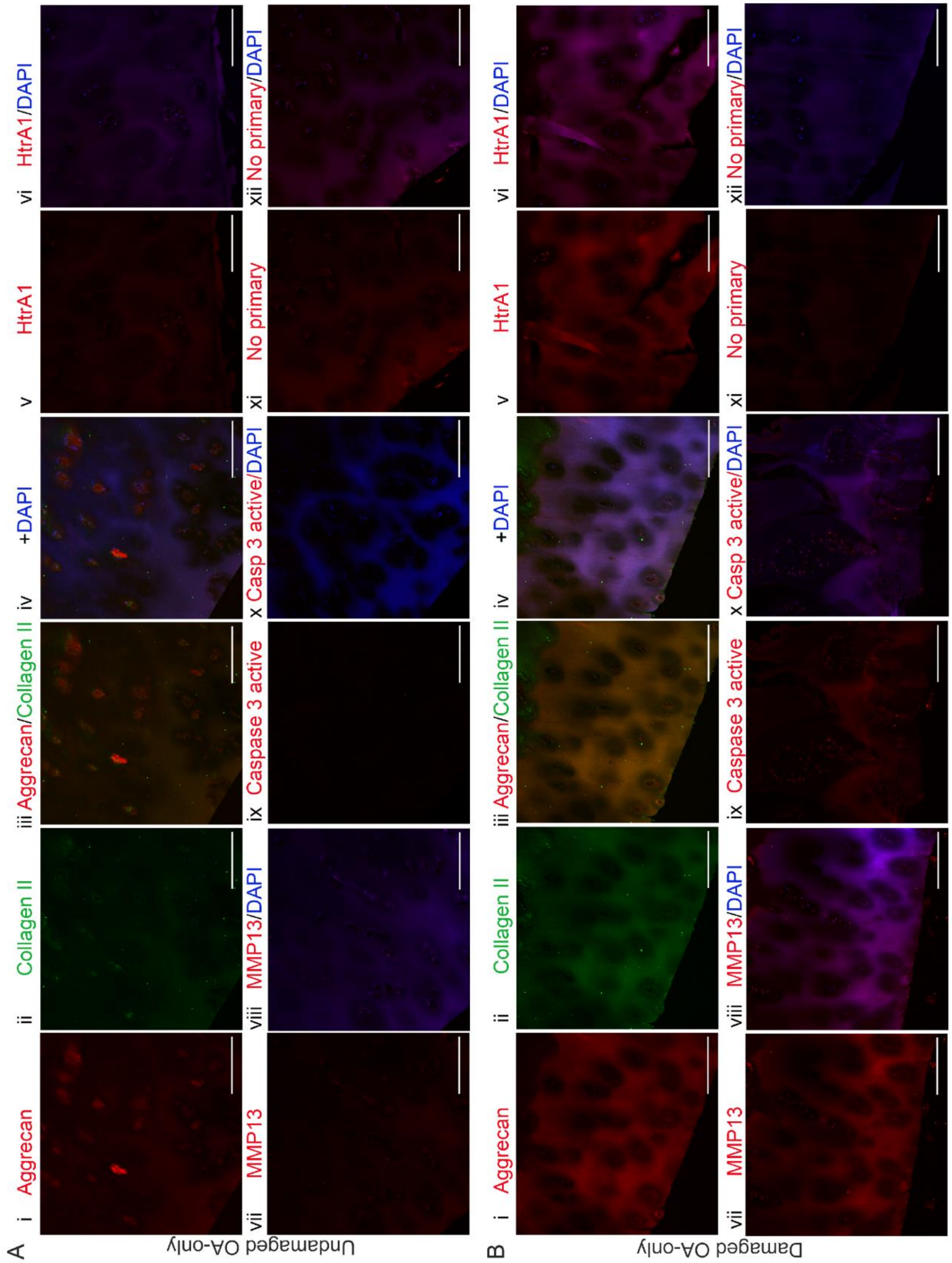

Supplementary Fig. S1: Confocal images of deep layer of cartilage for OA-only samples shown in Fig.3. A) Panel showing an undamaged OA-only sample stained for Aggrecan (i, red), Collagen II (ii, green), overlay (iii), overlay with DAPI (iv), HtrA1 (v, red), HtrA1 + DAPI (vi), MMP13 (vii, red), MMP13 + DAPI (viii), Caspase-3 active (ix, red), Caspase-3 active + DAPI (x), No primary control (xi, red), and No primary control + DAPI (xii). Only Aggrecan and Collagen II were co-stained on the same sample. The images for the other antibodies' staining are in equivalent areas. B) Same array of panels for a damaged OA-only sample. Scale bars are 200 µm. Images were taken with 20x objective.

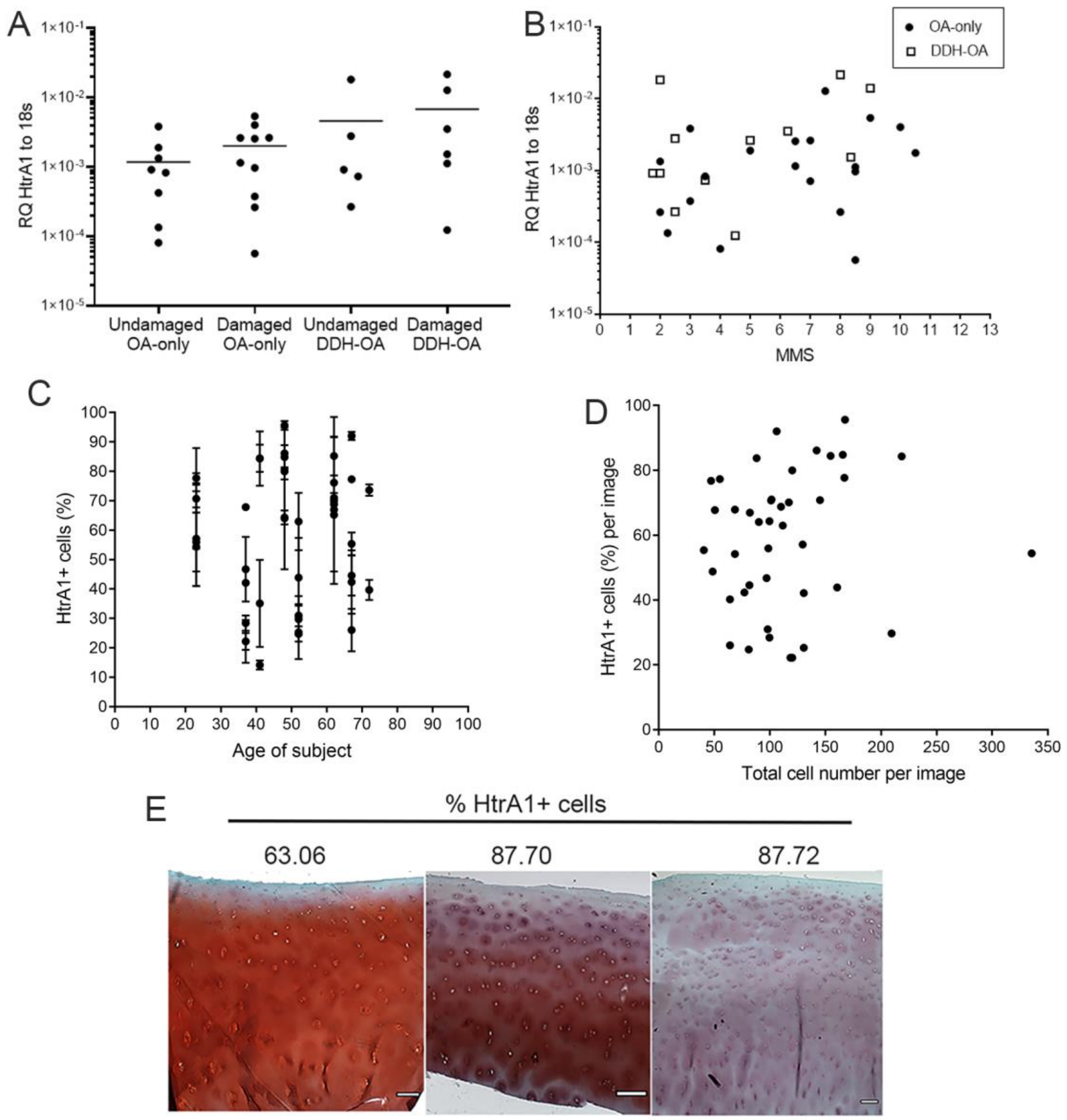

Supplementary Fig. S2: No apparent correlation between the percentage of HtrA1$^+$ cells and additional factors. A) Graph showing HtrA1 mRNA expression in the four groups studied. Values are relative quantity (RQ) to 18s. Scale is $\log_{10}$ for better display. Lines represent averages. There were no significant differences between groups. B) Graph showing no relationship between HtrA1 mRNA expression level and MMS. Results are shown as RQ to 18s and in $\log_{10}$ for better display. Pearson's r= 0.2721, p=0.2458, N= 20 for OA-only and r=-0.3822, p=0.2202, N= 12 for DDH-OA samples. C) Graph showing no relationship detected between % HtrA1$^+$ cells and age of subject. Results presented as mean per patient per condition (undamaged and damaged) with SD. N= 43 images from 9 patients. D) Dot plot showing no relationship detected between the percentage of HtrA1$^+$ cells and total cell number per image. Results presented as mean of 2 blind counts per patient with SD. N= 43 images from 9 patients. Error bars were omitted for clarity. E) Representative images of DDH-OA samples stained for proteoglycan content with Safranin-O. The percentage of HtrA1$^+$ cells is shown above each image. Scale bars are 200 µm.

Supplementary Fig. S3

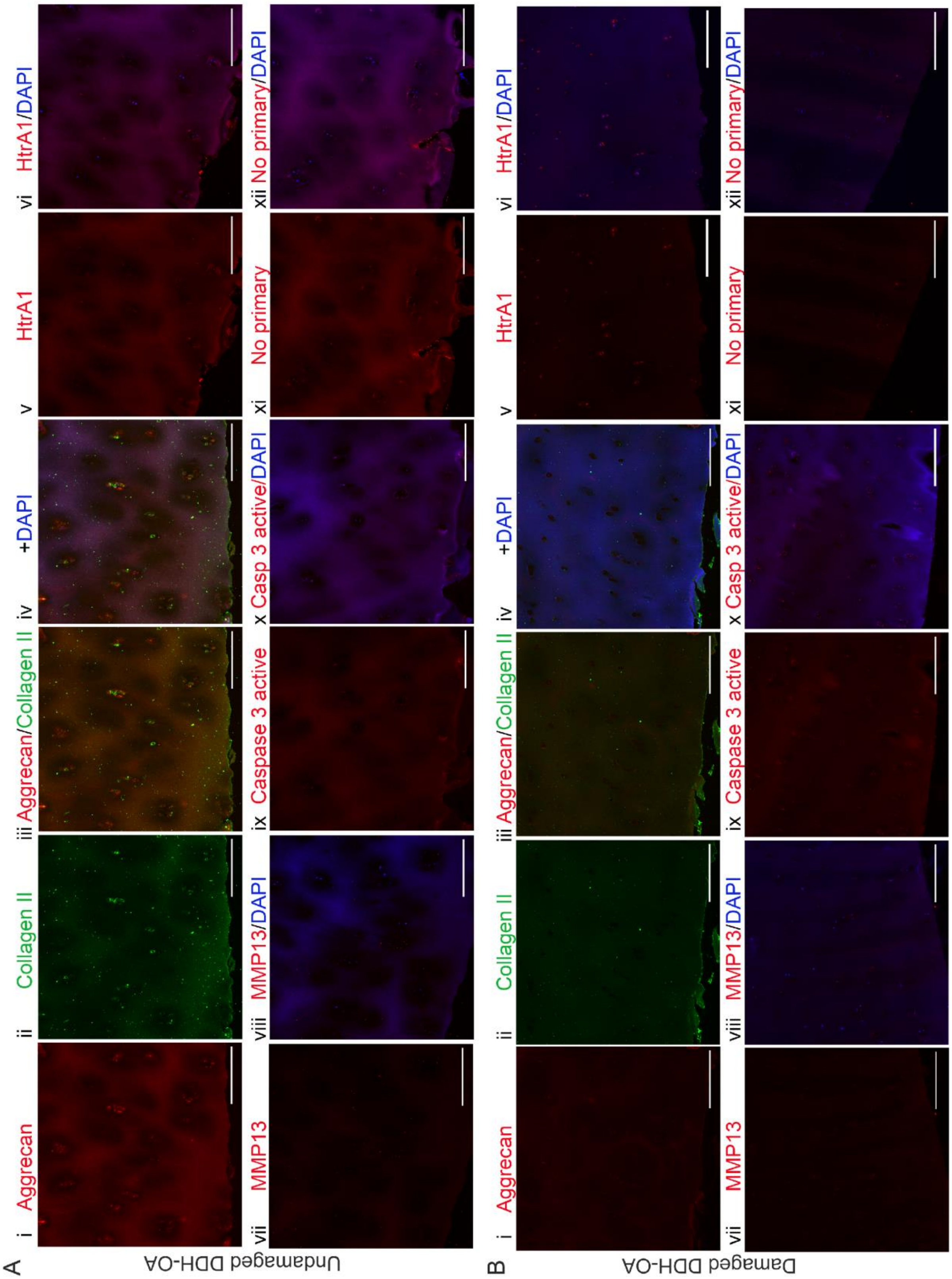

Supplementary Fig. S3: Confocal images of deep layer of cartilage for DDH-OA samples shown in Fig. 5. A) Panel showing an undamaged DDH-OA sample stained for Aggrecan (i, red), Collagen II (ii, green), overlay (iii), overlay with DAPI (iv), HtrA1 (v, red), HtrA1 + DAPI (vi), MMP13 (vii, red), MMP13 + DAPI (viii), Caspase-3 active (ix, red), Caspase-3 active + DAPI (x), No primary control (xi, red), and No primary control + DAPI (xii). Only Aggrecan and Collagen II were co-stained on the same sample. The images for the other antibodies' staining are in equivalent areas. B) Same array of panels for a damaged DDH-OA sample. Scale bars are 200 µm. Images were taken with 20x objective.
